# Reconsidering Surgical Intervention for Infection After Cranioplasty in Older Adults: A Case Report

**DOI:** 10.7759/cureus.109313

**Published:** 2026-05-20

**Authors:** Ryohei Saito, Ayumu Yamaoka, Asuka Takada, Yukinori Akiyama, Nobuhiro Mikuni

**Affiliations:** 1 Department of Neurosurgery, Sapporo Medical University School of Medicine, Sapporo, JPN; 2 Department of Plastic Surgery, Sapporo Medical University School of Medicine, Sapporo, JPN; 3 Department of Plastic, Reconstructive and Aesthetic Surgery, Nippon Medical School, Tokyo, JPN

**Keywords:** cognitive impairment, extensive scalp defect, japanese geriatrics, post-traumatic brain injury, surgical decision-making

## Abstract

Post-cranioplasty infection with scalp breakdown in older adults poses a difficult reconstructive problem, particularly when frailty and cognitive impairment may limit tolerance for staged procedures. Although debridement, infection control, and delayed cranioplasty are often considered standard management, the extent of reconstruction that should be pursued in elderly patients remains uncertain. We report the case of a woman in her late 80s who developed scalp loss with exposure of a synthetic cranial implant eight months after cranioplasty following surgery for acute subdural hematoma. On admission, head computed tomography showed no intracranial infection, while three-dimensional computed tomography angiography demonstrated preserved patency of the superficial temporal artery with residual blood supply around the defect. Because her advanced age, cognitive impairment, and the anticipated burden of staged reconstruction raised concern about tolerance for repeated procedures, treatment was directed toward infection control and durable wound closure without repeat cranioplasty. Removal of the implant revealed an epidural abscess, and thorough debridement was performed. Under the infected artificial dura, a well-formed fibrous capsule was identified and used for duraplasty. The scalp defect was reconstructed with a free latissimus dorsi musculocutaneous flap and split-thickness skin grafting. No recurrent infection developed, although postoperative delirium limited rehabilitation and functional recovery. This case highlights that delayed recognition of wound complications in cognitively vulnerable older adults can allow progression to extensive defects and that omission of cranioplasty may be a reasonable endpoint in selected patients when durable soft-tissue coverage and infection control take precedence over anatomical restoration.

## Introduction

Postoperative infection and extensive scalp defects after craniotomy or cranioplasty remain challenging complications [1,2]. In elderly patients, decreased skin integrity and impaired resistance to infection are common, often accompanied by frailty and cognitive vulnerability [3-5]. These factors increase the risk of postoperative complications and poor functional outcomes, including discharge to long-term care facilities [6]. In this setting, decisions regarding morphological reconstruction after cranioplasty failure present a significant clinical challenge [7]. When surgical interventions substantially affect functional independence, treatment priorities may shift from anatomical restoration to preservation of overall function. In geriatric neurosurgical care, guidance on reassessing such decisions according to clinical condition and functional prognosis remains limited. In this report, we present a case of postoperative infection and extensive scalp defects following cranioplasty after surgical treatment for an acute subdural hematoma in an elderly patient. We discuss treatment selection and decision-making strategies for post-cranioplasty infection in older adults.

## Case presentation

**Figure 1:**
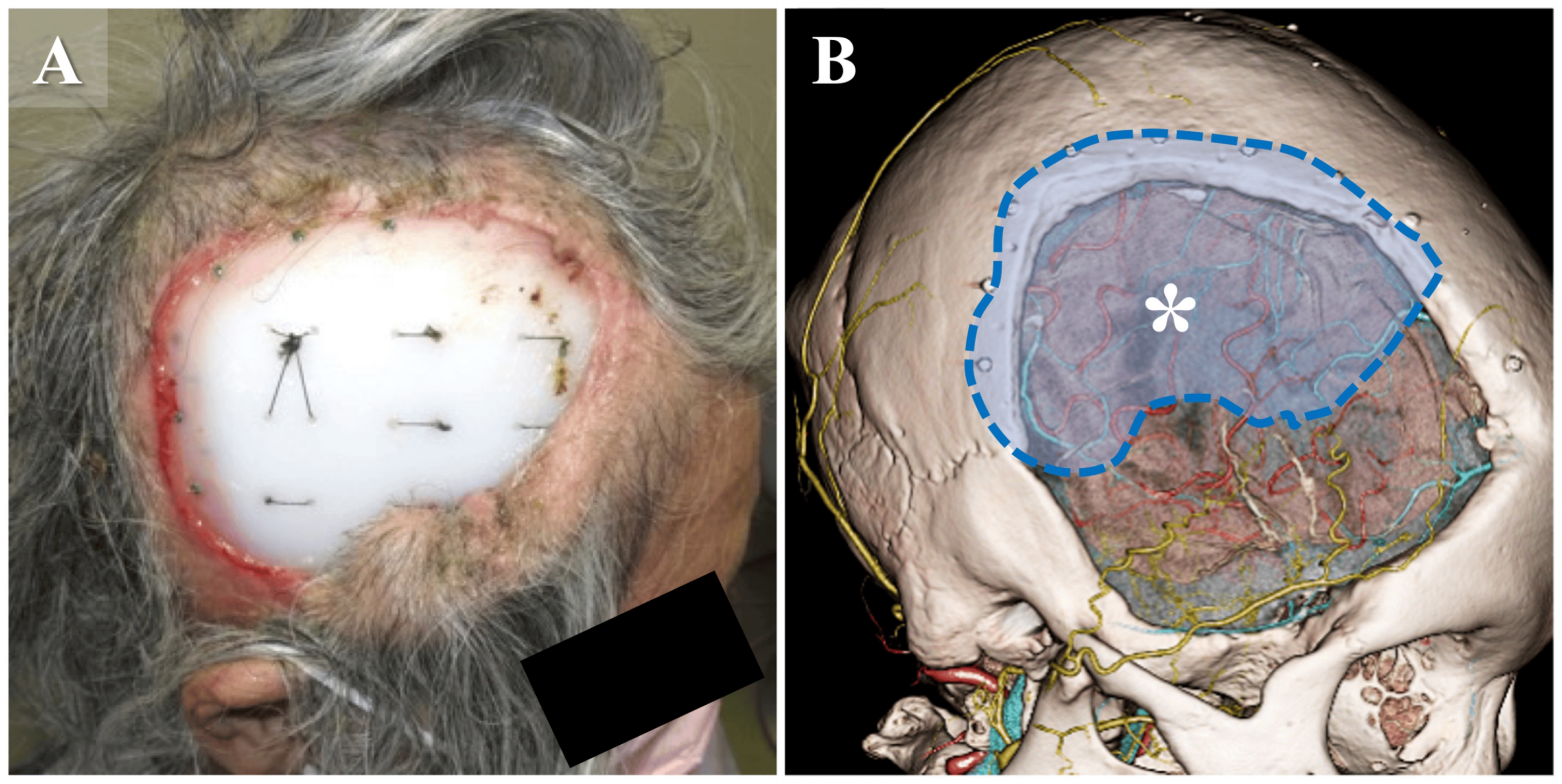
Preoperative findings on admission. (A) Preoperative appearance on admission showing a large scalp defect with exposure of the synthetic cranial implant. (B) Three-dimensional CT angiography demonstrating the scalp defect (white asterisk) and the superficial temporal and occipital arteries highlighted in yellow.

An elderly woman in her late 80s underwent craniotomy for hematoma evacuation and decompressive craniectomy at another hospital for a right acute subdural hematoma caused by a fall. Her postoperative course was initially uneventful, and autologous cranioplasty was performed one month later. However, postoperative hemorrhage occurred, necessitating hematoma evacuation and removal of the bone flap. Two months later, cranioplasty using a synthetic implant was performed, and she was discharged home. At an outpatient follow-up visit eight months after the cranioplasty, she reported wound discharge. Physical examination revealed a scalp defect with exposure of the synthetic implant (Figure 1A), and she was referred to our department for multidisciplinary management.

**Figure 2:**
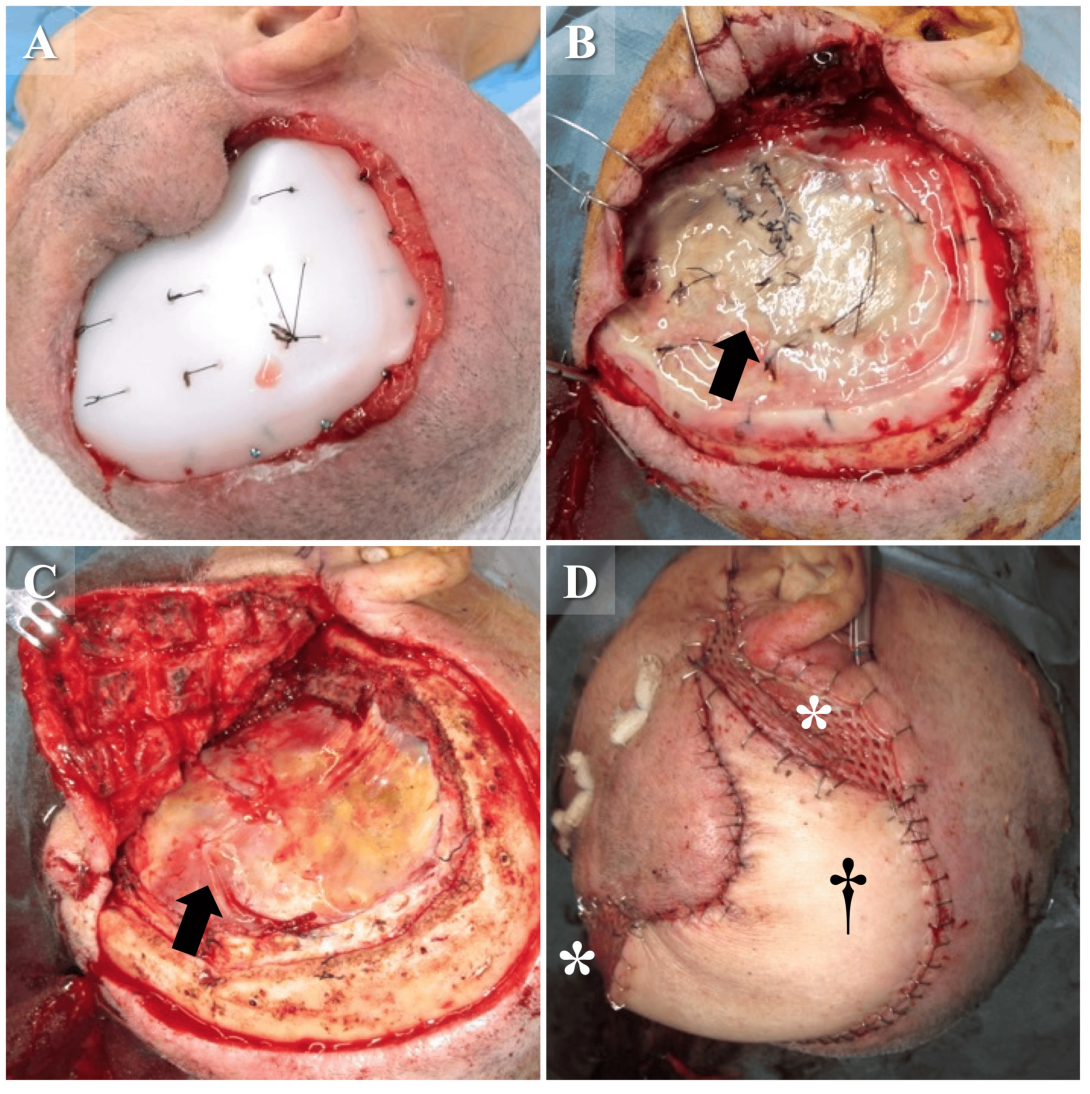
Intraoperative photographs. (A) Exposure of the synthetic cranial implant through the scalp defect. (B) Epidural abscess (black arrow) identified after removal of the polyethylene cranial implant. (C) Fibrous capsule (black arrow) observed beneath the dura after debridement and removal of the artificial dura mater. (D) Reconstruction of the scalp defect with a free latissimus dorsi musculocutaneous flap (black dagger) and split-thickness skin grafting (white asterisk).

On admission, her level of consciousness was Glasgow Coma Scale 14 (E4V4M6), and no other focal neurological deficits were identified. Cognitive impairment had been noted at the referring hospital, where the Mini-Mental State Examination score was 18/30 [8]. Laboratory studies showed a white blood cell count of 5,300/µL and a C-reactive protein level of 1.53 mg/dL (Table 1). Head computed tomography demonstrated no evidence of subdural hemorrhage or intracranial infection. Three-dimensional computed tomography angiography demonstrated that residual blood supply from the superficial temporal and occipital arteries persisted around the defect despite the scalp loss (Figure 1B). Definitive management would typically require debridement of the infected tissue and removal of the implant, reconstruction of the scalp defect, followed by antibiotic therapy and delayed cranioplasty. However, her advanced age and cognitive impairment suggested limited tolerance for such staged reconstructive procedures. We therefore decided not to proceed with cranioplasty and instead focused on wound closure and infection control. Reconstruction with a free flap was planned in collaboration with the plastic surgery team. Intraoperatively, removal of the polyethylene cranial implant revealed an epidural abscess (Figures 2A-2B), which was thoroughly debrided. After removal of the expanded polytetrafluoroethylene artificial dura mater, a fibrous capsule was observed in the subdural space (Figure 2C). The capsule appeared sufficiently robust for dural closure, and we therefore elected to use it for duraplasty. Subsequently, a free latissimus dorsi musculocutaneous flap was transferred, with microvascular anastomosis to the superficial temporal artery and deep temporal vein, followed by split-thickness skin grafting by the plastic surgery team (Figure 2D). Intravenous cefazolin was administered as empirical therapy targeting methicillin-susceptible *Staphylococcus aureus*.

**Table 1:** Laboratory findings on admission. ALT: alanine transaminase; APTT: activated partial thromboplastin time; AST: aspartate aminotransferase; LDH: lactate dehydrogenase; FDP: fibrin degradation products; PT-INR: prothrombin time-international normalized ratio

Laboratory test	Result	Reference range
Hematology		
White blood cell count (×10^3^/μL)	5.3	3.3-8.6
Neutrophils count (×10^3^/μL)	3.5	
Red blood cell count (×10^6^/μL)	3.62	3.86-4.92
Hemoglobin (g/dL)	10.7	11.6-14.8
Hematocrit (%)	34.6	35.1-44.4
Platelet count (×10^3^/μL)	326	158-348
Electrolytes/Metabolic Panel		
Sodium (mmol/L)	145	138-145
Potassium (mmol/L)	4.2	3.6-4.8
Chloride (mmol/L)	106	101-108
Calcium (mg/dL)	8.9	8.8-10.1
Glucose (mg/dL)	116	73-109
HbA1c (NGSP) (%)	6.1	4.6-6.2
Glycoalbumin (%)	17.7	11.0-16.0
Renal Function		
Blood urea nitrogen (mg/dL)	14	8-20
Creatinine (mg/dL)	0.62	0.46-0.79
Liver/Protein Profile		
Total protein (g/dL)	6.9	6.6-8.1
Albumin (g/dL)	3.4	4.1-5.1
Total bilirubin (mg/dL)	0.4	0.4-1.5
AST (U/L)	11	13-30
ALT (U/L)	4	7-23
LDH (U/L)	142	124-222
Creatine kinase (U/L)	39	41-153
Inflammatory Markers		
C-reactive protein (mg/dL)	1.53	0.00-0.14
Coagulation		
PT-INR	1.04	0.85-1.15
APTT (sec)	29.8	24.0-34.0
FDP (μg/mL)	<2.0	<5
D-dimer (μg/mL)	0.8	<1.0

**Figure 3:**
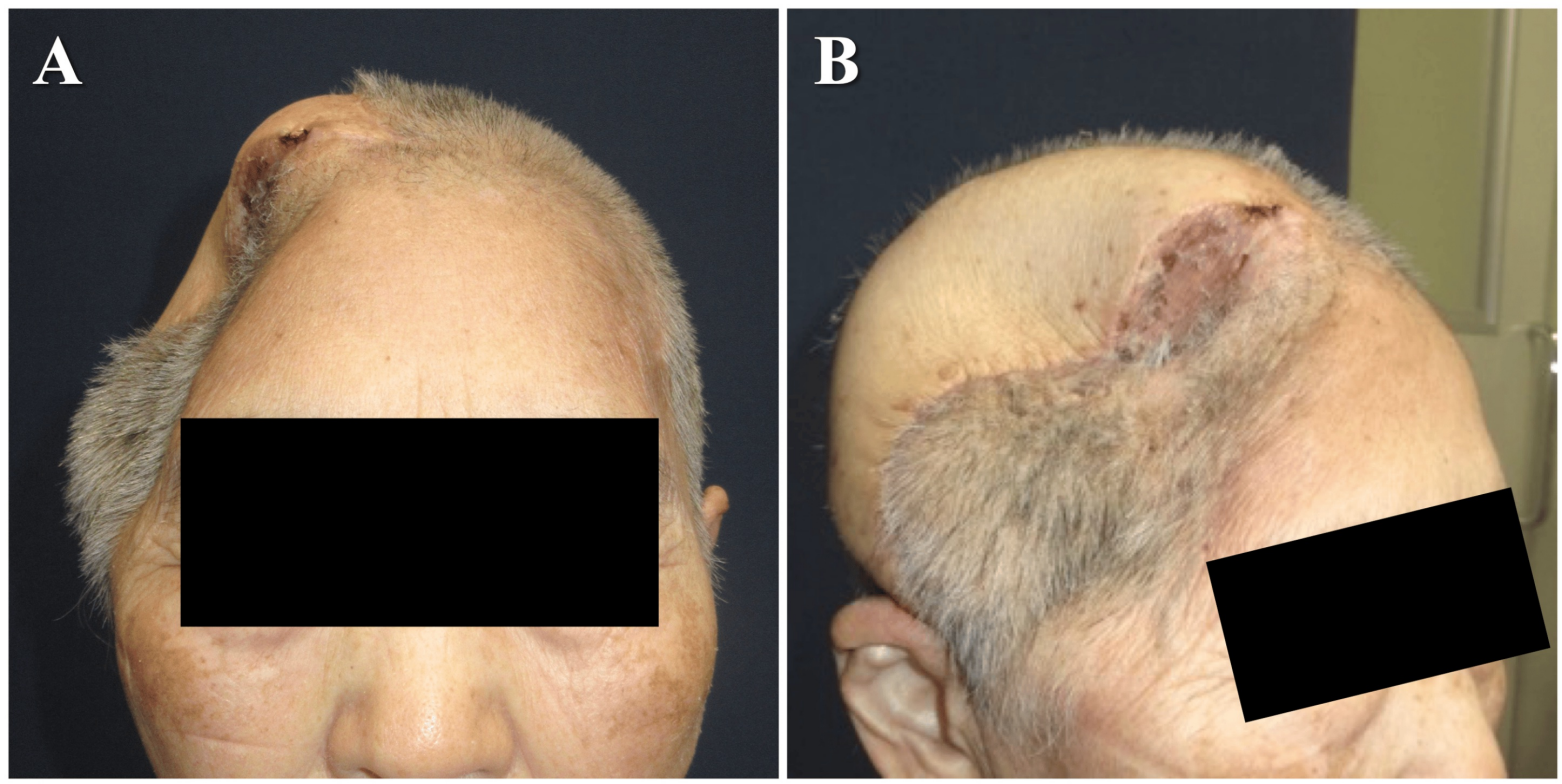
Postoperative appearance at one month. (A) Mild flap depression without clinical features suggestive of sinking skin flap syndrome. (B) Good flap condition with no signs of recurrent infection.

Based on the local wound findings, the absence of systemic inflammatory response, and the lack of radiological evidence of intracranial infection, antibiotic therapy was completed after one week. At one month after surgery, the free flap remained viable, and no signs of recurrent infection were observed (Figures 3A-3B). However, postoperative delirium with increased irritability developed, which limited early mobilization. Active rehabilitation was initiated one week after surgery. Due to disuse syndrome, the patient was transferred to another hospital with a modified Rankin Scale score of 4. After the transfer, she remained wheelchair-dependent. At 14 months after surgery, the patient was living at home under the care of her family, primarily using a wheelchair for mobility. No recurrence of wound infection was observed.

## Discussion

Two clinical insights were derived from this case. First, in elderly patients, cognitive impairment can delay recognition of early local infection, resulting in significant defects requiring complex reconstruction. Second, in the context of post-cranioplasty infection in elderly patients, avoiding cranioplasty may be an acceptable surgical option depending on patient factors and functional considerations.

Once infection, implant exposure, and tissue loss are established, durable scalp and soft-tissue coverage becomes pivotal for infection control. Reconstructive options include local flaps and free tissue transfer and are selected based on defect size, surrounding tissue condition, exposed structures, dead space, and patient-related factors [9,10]. Large defects, thin or scarred scalp, and residual dead space often necessitate free tissue transfer [2,9]. Such reconstructive burden may result in postoperative functional decline, raising concerns about the suitability of the surgical strategy, particularly in older adults. Early recognition of local wound problems is therefore essential. In the present case, because no routine outpatient follow-up was scheduled after discharge home, an extensive scalp defect with implant exposure remained unrecognized for a prolonged period. The extent of the defect made local reconstruction impractical, and free tissue transfer became the only realistic reconstructive option. Complications related to cranial implants, including infection and implant exposure, may occur long after surgery, underscoring the importance of continued follow-up after cranioplasty [11]. Cognitive decline may reduce both symptom recognition and self-care, including personal grooming, thereby limiting attention to scalp hygiene and wound changes [12]. The absence of structured follow-up and impaired cognition may both have contributed to delayed recognition and progression of the wound. This case supports structured outpatient follow-up in cognitively vulnerable older adults after cranioplasty, with caregiver engagement and attention to wound and personal care.

In post-cranioplasty infection, reconstruction is typically performed either immediately at the time of debridement (one stage) or after infection control (two stage) [13], while omission of cranioplasty may also be considered in carefully selected patients. From a decision-making perspective, reconstructive cranioplasty and omission of cranioplasty involve different trade-offs. Although reconstructive cranioplasty may contribute to long-term functional recovery and quality of life [14], it often requires staged procedures and a prolonged treatment course. It also carries a continuing risk of implant-related complications or reconstructive failure [15,16]. In contrast, omission of cranioplasty reduces cumulative operative burden and simplifies infection control. The absence of cranial restoration may also lead to delayed issues, such as contour deformity, reduced cranial protection, or sinking skin flap syndrome [17]. Continued surveillance and reassessment for delayed cranioplasty should therefore be considered. These considerations may be particularly relevant in frail older adults, in whom physiological reserve, rehabilitation tolerance, and cumulative surgical stress require careful consideration [5,6,15]. In the present case, both local and patient factors raised concern about staged reimplantation. The local factors included compromised scalp and soft-tissue quality, extensive tissue loss, and dead space. The patient factors included cognitive vulnerability and limited reserve. These factors suggested poor tolerance for repeated procedures and prolonged recovery. Under these conditions, durable soft-tissue coverage and infection control were prioritized over anatomical cranial reconstruction. This case suggests that omission of cranioplasty may be considered when local tissue conditions and patient vulnerability make staged reimplantation high risk.

A further consideration is intraoperative dural reconstruction after removal of the infected artificial dura. Options include vascularized pedicled flaps, autologous free grafts, or alternative substitutes, depending on the degree of contamination and defect characteristics. Pericranial flap-based reconstruction provides vascularized tissue that may enhance resistance to infection after adequate debridement. Its limitations include insufficient surface area for extensive defects and the need for additional dissection and operative time [18]. Autologous free grafts, such as fascia lata, can provide broad coverage for large defects. Their disadvantages include donor-site morbidity and potential delay in postoperative mobilization, particularly in frail older adults. In our patient, the extensive scalp defect precluded the use of a pericranial flap. Intraoperatively, we identified a well-formed fibrous capsule in the subdural space. To avoid donor-site morbidity, including pain and potential gait impairment associated with fascia lata harvesting, we elected to use this capsule as an autologous dural substitute. When a well-formed capsule is present and in the absence of cerebrospinal fluid leakage, preservation or utilization of the capsule as a dural substitute has been reported as a feasible option in selected infected cases [19]. In cognitively impaired older adults, this strategy may represent a pragmatic option by minimizing additional surgical morbidity. However, such an approach does not guarantee favorable functional recovery, as postoperative delirium may still impede early rehabilitation. The risk of infection recurrence and the long-term durability of using a fibrous capsule have not been well established [20]. Therefore, this approach should be regarded as a selective intraoperative option rather than a standard substitute for formal dural reconstruction.

This report has important limitations. As this is a single case report without standardized baseline frailty and cognitive assessments, the findings should be interpreted carefully. The favorable local outcome may also reflect case-specific surgical and patient factors. Therefore, this case should not be interpreted as supporting the superiority or broad generalizability of omission of cranioplasty over staged reconstruction.

## Conclusions

In older adults with post-cranioplasty infection and extensive scalp defects, treatment goals should be reassessed as local tissue conditions and patient vulnerability evolve. Cognitive impairment during postoperative follow-up may delay recognition of early infection and allow wound problems to progress to defects requiring complex reconstruction; therefore, structured outpatient surveillance with caregiver engagement is essential. In selected patients, choosing no cranioplasty can be a reasonable endpoint to prioritize durable infection control and stable soft-tissue coverage while minimizing cumulative surgical stress.
